# Not all dimers are equal

**DOI:** 10.7554/eLife.106980

**Published:** 2025-05-02

**Authors:** Jeet H Patel, Mary C Mullins

**Affiliations:** 1 Department of Cell and Developmental Biology, University of Pennsylvania Philadelphia United States

**Keywords:** cell signaling, BMP4, prodomain, developmental biology, human mutations

## Abstract

Disease-causing mutations in the signaling protein BMP4 impair its secretion, but only when it is made as a homodimer.

**Related research article** Kim H, Sanchez M, Silva J, Schubert HL, Dennis R, Hill CP, Christian JL. 2025. Mutations that prevent phosphorylation of the BMP4 prodomain impair proteolytic maturation of homodimers leading to lethality in mice. *eLife* **14**:RP105018. doi: 10.7554/eLife.105018.

Various signaling pathways guide the organization of cells and tissues in the body. Among these is the Bone Morphogenetic Protein (BMP) pathway, which plays a vital role in instructing early development, maintaining the cardiovascular system as well as forming and regenerating bones (Wang et al., 2014).

BMP ligands bind to receptor complexes on recipient cells, triggering a signaling cascade that alters gene expression. Thirteen different BMP ligands exist, which can be secreted either as homodimers (two identical proteins) or heterodimers (two different proteins); BMP4, for example, can form dimers with itself or with BMP7. Previous work has revealed exclusive roles for BMP heterodimers in tissue patterning (Shimmi et al., 2005; Tajer et al., 2021), including BMP2/BMP7 and BMP4/BMP7 heterodimers being critical for signaling in mouse embryogenesis, notably during heart development (Kim et al., 2019). However, definitive in vivo contexts that require only homodimers have been less clearly identified.

Mutations in certain BMP ligands and receptors can lead to developmental defects and other disease conditions. Often, such mutations cause changes in a single amino acid that does not fully abrogate protein function. Understanding how mutations affect BMP signaling can provide new insights into how the pathway is regulated, and may help identify better ways to treat patients. Now, in eLife, Jan Christian and co-workers from the University of Utah – including joint first authors Hyung-seok Kim, Mary Sanchez, and Joshua Silva – report how two clinically relevant mutations in the gene for BMP4 affect BMP signaling (Kim et al., 2025). These mutations – BMP4^S91C^ and BMP4^E93G^ – each change a single amino acid in the prodomain, a part of the protein that is normally removed before secretion (Cui et al., 2001; Figure 1). Both mutations are associated with colorectal cancer as well as developmental defects such as a cleft lip, spina bifida and improper kidney formation (Weber et al., 2008; Bakrania et al., 2008).

**Figure 1.**
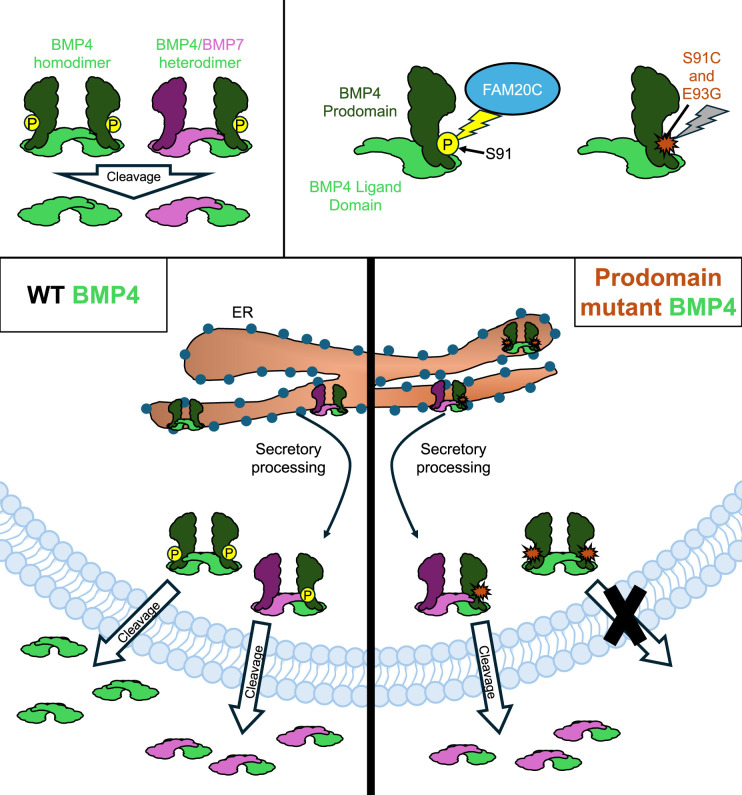
How two mutations associated with developmental defects impact BMP4 secretion. The signaling protein BMP4 (green) is secreted either as a homodimer made up of two identical BMP4 proteins, or a heterodimer comprised of BMP4 and another BMP protein, such as BMP7 (pink; top left panel). An enzyme called FAM20C (blue) adds a phosphoryl group (yellow circle) to the prodomain of BMP4 before it is secreted (top right panel). However, two mutations associated with development defects – S91C and E93G – block this phosphorylation (indicated by a red marker), leading to disruptions in the BMP signaling pathway. In wild-type (WT) embryos, BMP4 homodimers and BMP4/BMP7 heterodimers are generated in the endoplasmic reticulum (ER; bottom left panel). They are then processed for secretion as they exit the ER and head towards the plasma membrane, where the prodomain is cleaved, allowing the dimer to be exported. Kim et al. found that the prodomain mutations S91C and E93G disrupt cleavage and secretion of BMP4 homodimers, but not BM4/BMP7 heterodimers (bottom right panel).

First, using embryos from the African clawed frog *Xenopus laevis*, Kim et al. set out to find how these mutations may impact signaling by BMP4 homodimers and BMP4/BMP7 heterodimers. The team expressed wild-type BMP4 homodimers and BMP4/BMP7 heterodimers in regions of the frog embryo where BMP signaling is normally inactive. Both wild-type ligands were able to activate the pathway. However, when BMP4^S91C^ or BMP4^E93G^ was expressed, the signalling activity of the homodimer, but not the BMP4/BMP7 heterodimer, was reduced. This suggests that the mutations selectively disrupt homodimer function while preserving heterodimer activity.

The team then leveraged this finding to identify developmental processes where BMP4 homodimers are specifically required. They created mice that expressed BMP4^S91C^ or BMP4^E93G^. Embryos carrying the S91C mutation died 10–12 days into gestation, while pups carrying the E93G mutation had craniofacial defects and died shortly after birth. These findings support the idea that BMP4 homodimers signal at specific stages of development in the mouse embryo.

Finally, Kim et al. investigated how the two mutations could reduce BMP4 homodimer signaling. Both mutations reside in a highly conserved region of the prodomain that is phosphorylated by the kinase FAM20C and may be important for protein folding, cleavage or secretion (Tagliabracci et al., 2015). The team found that neither BMP4^S91C^ nor BMP4^E93G^ hindered protein folding or exit from the endoplasmic reticulum. However, both mutations reduced prodomain cleavage and the amount of ligand secreted from cells. Therefore, Kim et al. propose that FAM20C-mediated phosphorylation is critical for BMP4 homodimer secretion, though how exactly this modification facilitates prodomain cleavage and ligand release has not yet been elucidated.

These findings lay the groundwork for understanding how BMP4 homodimers and BMP4/BMP7 heterodimers differ in secretion and signaling across developmental contexts. A recent study showed that the prodomain of the ligand BMP2 is needed for secretion of BMP2/BMP6 heterodimers in *Xenopus* embryos but not in human liver endothelial cells (Chauhan et al., 2024), suggesting that specific prodomains regulate secretion in different cell types. The observation that BMP4/BMP7 heterodimers are not impacted by either the BMP4^S91C^ or BMP4^E93G^ mutation suggests that they use a different secretory mechanism than BMP4 homodimers.

It remains to be seen how mutations in the prodomain selectively disrupt homodimer secretion, and at which step in the cleavage process phosphorylation is required. Examining the cleavage states and secretion of the BMP4 mutant dimers, and how the presence of BMP7 affects these processes, may also illuminate how heterodimers are processed for signaling. Ultimately, these insights could inform the development of targeted therapies for diseases driven by specific BMP signaling components.
